# Population-level transcriptome sequencing of nonmodel organisms *Erynnis propertius *and *Papilio zelicaon*

**DOI:** 10.1186/1471-2164-11-310

**Published:** 2010-05-17

**Authors:** Shawn T O'Neil, Jason DK Dzurisin, Rory D Carmichael, Neil F Lobo, Scott J Emrich, Jessica J Hellmann

**Affiliations:** 1 Department of Computer Science and Engineering, University of Notre Dame, Notre Dame, IN, USA; 2 Department of Biological Sciences, University of Notre Dame, Notre Dame, IN, USA

## Abstract

**Background:**

Several recent studies have demonstrated the use of Roche 454 sequencing technology for *de novo *transcriptome analysis. Low error rates and high coverage also allow for effective SNP discovery and genetic diversity estimates. However, genetically diverse datasets, such as those sourced from natural populations, pose challenges for assembly programs and subsequent analysis. Further, estimating the effectiveness of transcript discovery using Roche 454 transcriptome data is still a difficult task.

**Results:**

Using the Roche 454 FLX Titanium platform, we sequenced and assembled larval transcriptomes for two butterfly species: the Propertius duskywing, *Erynnis propertius *(Lepidoptera: Hesperiidae) and the Anise swallowtail, *Papilio zelicaon *(Lepidoptera: Papilionidae). The Expressed Sequence Tags (ESTs) generated represent a diverse sample drawn from multiple populations, developmental stages, and stress treatments.

Despite this diversity, > 95% of the ESTs assembled into long (> 714 bp on average) and highly covered (> 9.6× on average) contigs. To estimate the effectiveness of transcript discovery, we compared the number of bases in the hit region of unigenes (contigs and singletons) to the length of the best match silkworm (*Bombyx mori*) protein--this "ortholog hit ratio" gives a close estimate on the amount of the transcript discovered relative to a model lepidopteran genome. For each species, we tested two assembly programs and two parameter sets; although CAP3 is commonly used for such data, the assemblies produced by Celera Assembler with modified parameters were chosen over those produced by CAP3 based on contig and singleton counts as well as ortholog hit ratio analysis. In the final assemblies, 1,413 *E. propertius *and 1,940 *P. zelicaon *unigenes had a ratio > 0.8; 2,866 *E. propertius *and 4,015 *P. zelicaon *unigenes had a ratio > 0.5.

**Conclusions:**

Ultimately, these assemblies and SNP data will be used to generate microarrays for ecoinformatics examining climate change tolerance of different natural populations. These studies will benefit from high quality assemblies with few singletons (less than 26% of bases for each assembled transcriptome are present in unassembled singleton ESTs) and effective transcript discovery (over 6,500 of our putative orthologs cover at least 50% of the corresponding model silkworm gene).

## Background

Although the costs of genome sequencing have declined dramatically, full genome sequencing efforts are still impractical for many nonmodel species. In such cases, transcriptome sequencing provides a greatly informative and cost effective alternative [1,2]. Expressed Sequence Tag (EST) sequencing has been used in a variety of species for Single Nucleotide Polymorphism (SNP) discovery [3], gene discovery and annotation [4-7], and expression analysis [8-10].

While previous studies relied extensively on available genome or transcript data generated by Sanger EST sequencing, more recent results have used 454 technology to perform *de novo *assembly of transcriptomes. In 2008, Vera et al. sequenced ESTs of *Melitaea cinxia *using 454 GS20 technology, producing 108,297 contigs and singletons (ESTs which would not assemble with others), or "unigenes," representing an estimated 50% of the transcriptome [11]. Novaes et al. and Cheung et al. in the same year reported 454 EST assemblies for *Eucalyptus grandis *[12] and the plant pathogen, *Pythium ultimum *[13]. In 2009, Meyer et al. assembled the transcriptome of larval coral, *Acropora millepora*, to an average contig coverage of 5× [14], and Roeding et al. assembled the the transcriptome for the Emperor Scorpion, *Pandinus imperator*, to an average contig coverage of 9× [15], the highest of 454 transcriptome studies to date. These assemblies reinforce previous results that suggest 454 EST sequencing produces evenly covered transcripts with error rates mitigated by deep coverage [16].

Other published Lepidopteran EST projects include those for wing discs of adult *Heliconius erato *[17] and foreleg tarsi of *Papilio xuthus *[18]; both used Sanger based sequencing. In this paper, we present *de novo *larval full-body transcriptome assemblies for two butterflies: the Propertius Duskywing, *Erynnis propertius *(Lepidoptera: Hesperiidae), and the Anise Swallowtail, *Papilio zelicaon *(Lepidoptera: Papilionidae).

### Study Species

*E. propertius *is in the family Hesperiidae (Lepidoptera), a distinct branch of the butterflies called "skippers." *P. zelicaon *is in the family Papilionidae (Lepidoptera) and is more closely related to all other butterflies than to any skipper. *Erynnis propertius *and *P. zelicaon *co-occur in coastal, oak (Quercus) habitats containing native wildflowers that range from Baja California, Mexico northward into southwestern British Columbia [19,20]. *Erynnis propertius*, an oak specialist, is restricted to this range, whereas *P. zelicaon *also occurs further eastward and northward in the Rocky Mountains feeding on plants in the family *Apiaceae *[21].

Previous studies suggested that these species are differentiated across their range with populations at the northern range boundary being diverged from more central populations [22,23]. Zakharov and Hellmann [22] suggested that these differences could allow local adaptation of northern populations, possibly to local climatic conditions, and that these local adaptations could undermine the assumption that northern populations will increase under climate warming. Hellmann et al. [24] and Pelini et al. [25] investigated this possibility with a series of translocation experiments and found greater evidence for local adaptation of northern populations in *E. propertius *than *P. zelicaon*. Pelini et al. [25] also found that climate affects fitness on alternate host plants, switching the relative value of host species under different climate treatments in *P. zelicaon*.

Before we performed 454 sequencing, there were no genetic data for these species with the exception of microsatellites [26,27], mitochondrial genes, and select genes identified in other *Papilio *(e.g. [28,29]). When compared to other transcriptomes or genomes of Lepidoptera that have been entirely sequenced, *E. propertius *and *P. zelicaon *can offer new insights into the systematics and genomics of a group that has been widely recognized for its utility in ecology and evolutionary biology [30]. In addition to advancing comparative genomics in Lepidoptera, sequencing the transcriptomes of two co-occurring species with known ecology offers many future research opportunities in ecology and ecoinformatics.

The mRNA of whole larvae were extracted for sequencing for two reasons: 1) because larvae are a key bottleneck in the population dynamics and fitness of individual butterflies [31-35] and 2) to complement previous studies by our group that measure larval fitness under differing climatic conditions [24,25]. The immediate aim of transcriptome sequencing was construction of a microarray enabling comparison of transcribed genes under alternate climate treatments and of populations from differing geographic locations.

Adult females were collected from 4 and 2 populations of *E. propertius *and *P. zelicaon*, respectively, near the latitudinal center of the species' distributions (within 50 km of Medford, Oregon). In total, 53 larvae of *E. propertius *and 61 larvae of *P. zelicaon *were pooled prior to sequencing, representing a minimum of 11 and a maximum of 48 wild mothers in *E. propertius *and exactly 5 wild mothers for *P. zelicaon*. To build a robust microarray for the study of larval biology, steps were taken to maximize gene discovery within the larval stage. Multiple larval instars were sampled, and individuals were exposed to a variety of stress and host plant treatments to elicit genes important in the life history of larvae (see Methods).

## Results

### Sequencing and Assembly

Half of one picotiter plate of a 454 FLX sequencing run generated 416,689 ESTs of *E. propertius*. Reads were cleaned and vector trimmed with standard SeqClean [36] protocol (see Methods). In total, 397,230 (95%) *E. propertius *ESTs passed the cleaning process, with an average length 431 bp and median length 458 bp. 432,343 (95%) ESTs of *P. zelicaon *passed cleaning, having an average length of 401 bp and median length 422 bp. These data are publicly available at the NCBI Sequence Read Archive (see Methods).

We ran the cleaned EST datasets through CAP3 as well as the Celera Assembler, experimenting with parameter settings for each (see Methods). Using default parameter settings, CAP3 [37] produced fairly large assemblies--24.3 Mbp for *E. propertius*. Although we wish to avoid collapsing paralogs, large assemblies indicate separately assembled alleles [38]. Using custom parameters for CAP3, we reduced assembly sizes somewhat, but this still produced a large percentage of singletons (Table 1). Celera Assembler [39] produced a 20.6 Mbp assembly for *E. propertius *using the recommended settings for 454 FLX Titanium reads [40]. Using custom settings with the Celera Assembler produced assemblies with the smallest overall assembly size and highest average ortholog hit ratio, a measure of assembly quality (Table 1, see Annotation). The size of this final assembled *E. propertius *transcriptome (16.4 Mbp) is similar to that previously produced for the related butterfly species *M. cinxia *(approximately 16.1 Mbp [11]). While the final *P. zelicaon *assembly is somewhat larger (18.5 Mbp), differences in assembly size between assemblers and parameter sets were similar to those seen for *E. propertius*.

**Table 1:** Statistics for alternate assemblies. The last column indicates the average Ortholog Hit Ratio (a measure of assembly quality, see Annotation) of unigenes with hits to *Bombyx mori*.

	Contigs (Coverage)	Singletons	Assembly Size	BP in Singletons	*B. m*. Hits	
CAP3 Defaults (-p 90 -h 20)						

*E. p*.	15,444 (8.39×)	27,516	24.3 Mbp	46.0%	12,763	0.352
*P. z*.	17,983 (8.12×)	28,884	25.1 Mbp	43.0%	16,113	0.356

CAP3 -p 85 -h 90						

*E. p*.	11,687 (10.07×)	20,053	19.1 Mbp	42.4%	10,183	0.372
*P. z*.	13,332 (9.78×)	20,563	19.5 Mbp	38.9%	12,740	0.375

Celera Standard						

*E. p*.	23,263 (8.35×)	13,444	20.6 Mbp	21.3%	11,625	0.384
*P. z*.	25,822 (8.05×)	21,397	22.9 Mbp	24.4%	16,755	0.398

Celera Final						

*E. p*.	17,110 (10.05×)	10,934	16.4 Mbp	21.3%	9,393	0.402
*P. z*.	19,110 (9.63×)	18,847	18.5 Mbp	25.9%	12,485	0.412

**Figure 1:**
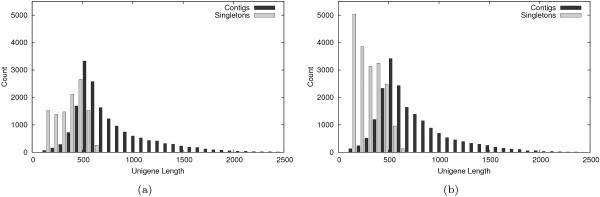
**Distributions of contig and singleton lengths for *E. propertius *(a) and *P. zelicaon *(b)**. Contigs longer than 2,500 bp (*n *= 34 for *E. propertius*, max length 3,737 bp, *n *= 26 for *P. zelicaon*, max length 3,621 bp) are not shown.

The custom Celera assembly for *E. propertius *resulted in 17,110 contigs and 10,934 singletons, for a total of 28,044 unigenes. Both the average contig length and average singleton length are noticeably larger than previous studies [11-14] at 753 bp and 324 bp, respectively. Cleaned *P. zelicaon *ESTs assembled into 19,110 contigs (average length 714 bp) and 18,847 singletons (average length 258 bp). The larger number of unassembled singletons for *P. zelicaon *may be due to mitochondrial rRNA sequences (see Clustering). Figure 1 shows the distributions of contig and singleton lengths for both species; other detailed assembly statistics also are found in Table 2.

**Table 2:** EST and final assembly statistics.

	Uncleaned Reads		Cleaned Reads		Contigs		Singletons		Unigenes		
	*n*	**bp**	*n*	**bp**	*n*	**bp**	*n*	**bp**	*n*	**bp**	*Median *bp
*E. p*.	416,689	424	397,230	431	17,110	753	10,934	324	28,044	586	502
*P. z*.	455,040	398	432,343	401	19,110	714	18,847	258	37,957	488	414

**Figure 2:**
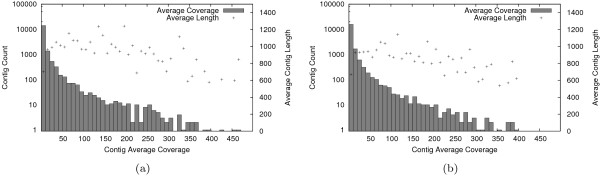
**Distribution of average contig read coverage for *E. propertius *(a) and *P. zelicaon *(b)**. On the *x *axis, contigs are grouped by average read coverage. On the *y *axis, the bars show the number of contigs in each coverage bin, and the points show the average length of contigs in each coverage bin.

Average (median) contig coverage was 10× (3.3×) for *E. propertius *and 9.6× (3.5×) for *P. zelicaon*. Figure 2 shows the contig coverage distributions for the two transcriptomes and the average sequence length for contigs within each coverage bin on a log scale. As expected and as found in previous studies [14,41], there was a positive correlation between contig length and the number of reads incorporated (data not shown). Figure 2 also shows that contigs with very high coverage (greater than 100×) tend to be shorter in length.

### Annotation

#### Bombyx mori, Gene Assembly Completeness

**Figure 3:**
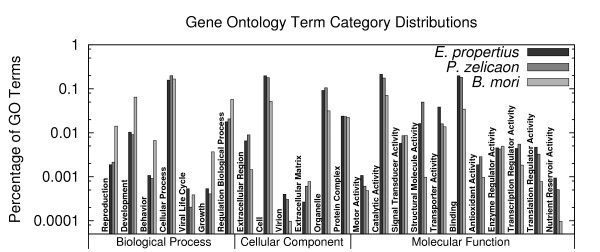
**Distribution of Gene Ontology terms for *E. propertius*, *P. zelicaon*, and *B. mori *(see Methods)**.

We compared the unigene sets to the predicted protein database for *Bombyx mori*, the silkworm, for which full genome data are available (GLEAN produced consensus gene set, SilkDB v2.0 [42]). This reference dataset contains 14,623 predicted *B. mori *proteins. Of the 28,044 *E. propertius *unigenes, 9,393 had BLASTX [43] (using a 1e-5 cutoff) hits to 7,866 unique *B. mori *predicted proteins. 5,289 unigenes hit more than one *B. mori *protein (average, 8.9, median, 2.0, amongst unigenes hitting at least one protein). 5,449 *B. mori *proteins were hit by more than one unigene (average, 10.6, median, 3.0, amongst proteins having at least one hit). Of the 37,957 *P. zelicaon *unigenes, 12,485 hit 8,359 unique *B. mori *predicted proteins; 6,518 hit more than one protein (average, 8.8, median, 2.0), and 5,883 proteins were hit by more than one unigene (average, 13.1, median, 3.0). Figure 3 shows the distribution of 24 categories for gene ontology terms, each categorized into three higher level categories, associated with the unigenes and the *B. mori *dataset (see Methods).

**Figure 4:**
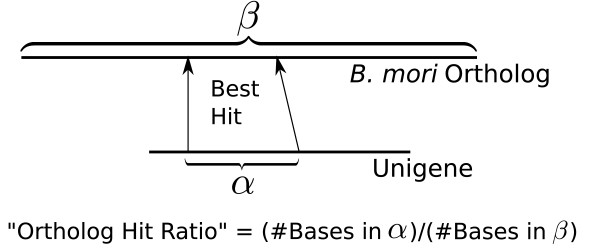
**The ortholog hit ratio describes the percentage of an ortholog "found" in a unigene by dividing the number of non-gap characters in the query hit by the length of the subject**.

For the purposes of this study, we consider each unigene and its best *B. mori *BLASTX hit to be orthologs, and we consider the hit region in the unigene to be a conservative estimator of the "putative coding region." Thus, we can compute the percentage of a unigene found by dividing the length of the putative coding region by the total length of the ortholog. This ratio, which we call the "ortholog hit ratio," is described in Figure 4. The assumption is that the unigene and its best hit are orthologs and not paralogs or some other mis-association. Using the conservative, BLAST based annotation to find putative coding regions, as opposed to non-comparative methods such as ESTScan [44], ensures that hit ratios are not overestimated.

The ortholog hit ratio gives an estimate on the amount of a transcript contained in each unigene. If there are relative insertions in best hit *B. mori *proteins, this will tend to lower ortholog hit ratios, whereas relative insertions in unigenes will artificially inflate ortholog hit ratios. Ortholog hit ratios greater than 1.0 likely indicate large insertions in unigenes.

**Figure 5:**
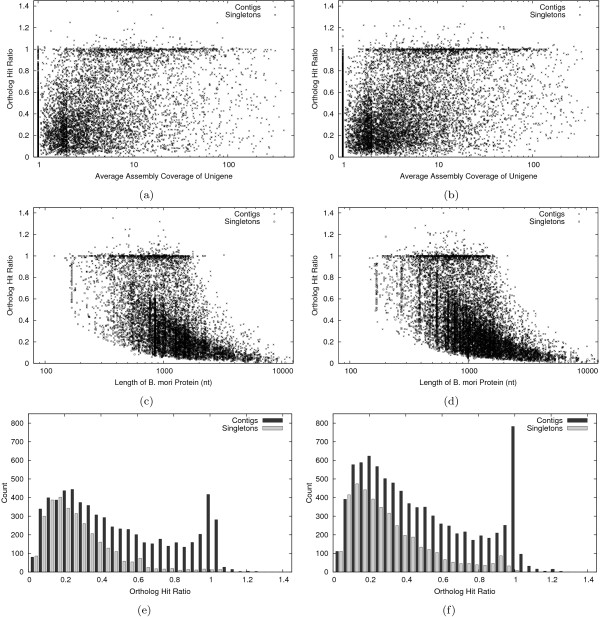
**Relationship between "ortholog hit ratio" (see Figure 4) and assembly coverage (a,b) as well as *B. mori *ortholog length (c, d)**. Figures on the left refer to *E. propertius*, figures on the right refer to *P. zelicaon*. Where this ratio is 1.0, the gene is likely fully assembled. Ratios greater than 1.0 can indicate insertions in unigenes. Overall distributions of ortholog hit ratios for contigs and singletons also shown (e,f).

Figures 5(a) and 5(b) show ortholog hit ratio in terms of assembly coverage of unigenes (which is 1.0 for singletons). For *E. propertius *contigs with less than the median assembly coverage of 3.3×, the average ortholog hit ratio was 0.35. For those with greater than median coverage, the average ratio was 0.56. The corresponding averages for *P. zelicaon *were 0.34 and 0.55, respectively. Thus, completeness of unigene assembly is partially governed by assembly coverage as expected.

Figures 5(c) and 5(d) relate ortholog hit ratio to the length of the *B. mori *ortholog. As found in other studies [11], completeness of gene discovery decreases as length of the gene increases. Vertical tracks in figures 5(c) and 5(d), comprised mostly of singletons, likely indicate regions of the genome that failed to assemble (see Clustering). Finally, figures 5(e) and 5(f) show the overall distributions of ortholog hit ratios for contigs and singletons. Overall, 1,413 of the 9,393 *E. propertius *unigenes having a hit to *B. mori *had ratio > 0.8, and 2,866 had ratio > 0.5. Of the 12,485 *P. zelicaon *unigenes with *B. mori *hits, 1,940 had ratio > 0.8 and 4,015 had ratio > 0.5.

#### Other Lepidoptera and Insecta

We also compared unigene sets to protein databases for *Drosophila melanogaster *(FlyBase, r5.22 [45]), containing 21,783 sequences and *Heliconius erato *(ButterflyBase, retrieved April, 2009 [46]), containing 8,790 sequences. *Drosophila melanogaster *proteins represent a well annotated insect transcriptome, and the *H. erato *database represents protein predictions based on tissue-specific Sanger EST data obtained from the wing discs of adults [46,47]. While this tissue-specific dataset is not as complete as the *D. melanogaster *protein dataset, comparison to the more related *P. zelicaon *and less closely related *E. propertius *reveals interesting differences.

5,688 *E. propertius *unigenes had BLASTX hits (1e-5 cutoff) to *H. erato *proteins. 7,497 had hits to *D. melanogaster *proteins. 11,082 *P. zelicaon *unigenes hit *H. erato *proteins, a much larger percentage (29.1% versus 20.2%), and 9,689 hit *D. melanogaster*.

**Figure 6:**
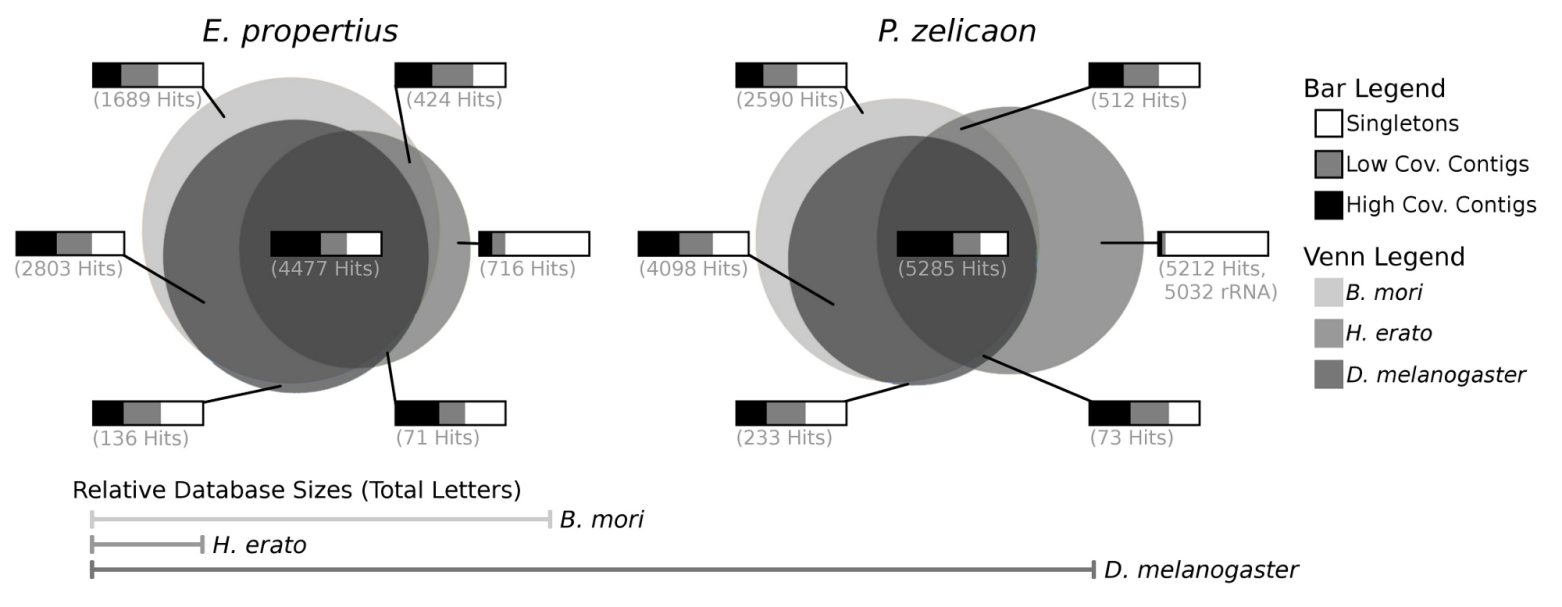
**BLAST unigene results relative to protein databases for *B. mori*, *H. erato*, and *D. melanogaster***. Note that these databases are not of equal size, and that the protein database for *H. erato *represents tissue-specific samples of adult wing discs [46,47]. Bars show the relative proportion of high coverage contigs (greater than median coverage), low coverage contigs (less than median coverage), and singletons for each Venn diagram area. Many of the hits for *P. zelicaon *unigenes which hit only to *H. erato *also hit to the mitochondrion 16S ribosomal RNA.

Figure 6 shows the number of unigenes with hits to one or more of the three protein databases. Venn diagram areas are scaled to represent percentages of the unigene sets. Although both species had a large number of hits to *H. erato*, this database is comparatively small as indicated by Figure 6.

The bars in Figure 6 show the relative proportion of high coverage contigs (greater than median coverage), low coverage contigs (less than median coverage), and singletons for each area in the Venn diagram.

Unigenes that hit to all three databases tend to have high coverage, while those that only hit the most related species (*H. erato*) tend to have low coverage or are singletons. 7,266 *E. propertius *singletons and 10,462 contigs with average coverage of 8× had no hits to these protein databases. For *P. zelicaon*, 9,871 singletons and 10,083 contigs with average coverage of 6.6× had no hits.

We also compared the unigene sets to recently sequenced whole larval *Melitaea cinxia *ESTs [11] and foreleg tarsi *Papilio xuthus *ESTs [18]. Although there is no protein, assembly, or annotation information publicly available for these datasets, we expect some similarity given phylogenetic distance. 9,979 *E. propertius *unigenes had TBLASTX hits (1e-5 cutoff) to 76,809 unique *M. cinxia *ESTs. 16,780 *P. zelicaon *unigenes had hits to 82,906 unique *M. cinxia *ESTs (out of 595,541). As expected, many more *P. zelicaon *unigenes had hits to *P. xuthus*--4,511 *E. propertius *unigenes hit 8,329 unique *P. xuthus *ESTs, while 16,492 *P. zelicaon *unigenes hit 13,043 unique *P. xuthus *ESTs (out of 16,802).

#### Clustering

To test whether incomplete assembly could account for the large proportion of singletons that hit only *H. erato *(Figure 6), we aggressively clustered unigenes by creating "association graphs" of best hits of unigenes to unigenes, unigenes to *H. erato *proteins, and unigenes to *B. mori *proteins. Unigenes in the same connected component were considered a cluster (see Methods). *E. propertius *unigenes produced 20,667 clusters, 19,037 of which contained only a single unigene. The largest *E. propertius *cluster contained 485 singletons (434 having hits only to *H. erato*) and 65 contigs (all of which hit only *H. erato*). *P. zelicaon *unigenes produced 21,530 clusters, 19,395 containing only a single unigene. For this species, a single very large cluster of 6,124 singletons and 209 contigs was produced. As with *E. propertius*, most of these singletons (4,832) and contigs (180) had hits only to *H. erato *proteins.

Most of the unigenes in the very large cluster for *P. zelicaon *were similar, though it appeared that a large amount of sequence diversity prevented their assembly into contigs. As mitochondrial genomes are frequently diverse in populations [22], we compared unigenes to *Papilio xuthus *mitochondrial genes and ribosomal RNAs (GenBank: EF621724). Of the 6,333 unigenes in the largest *P. zelicaon *cluster, 3 had a BLASTN hit to *P. xuthus *mitochondrial genes (*e *< 1e-5 cutoff) and 5,995 hit ribosomal RNA. We also identified 59 unigenes not present in the largest cluster that hit mitochondrial genes, and 1,275 that hit ribosomal RNA. All but a few ribosomal hits were to the 16S ribosomal RNA. Similar analysis of *E. propertius *unigenes revealed 43 hits to *P. xuthus *mitochondrial genes and 50 hits to ribosomal RNA, none of which occurred in the largest cluster.

To validate clustering results, we used TBLASTX (*e *< 1e-5 cutoff) to search for five single-copy genes from *B. mori*: CAD carbamoylphosphate synthase domain (GenBank:EU032656), PGD 6-phosphogluconate dehydrogenase (GenBank:NM 001047060), AATS alanyl-tRNA synthetase (GenBank:M55993), SNF sans fille (GenBank:DQ202313), and TPI triosephosphate isomerase (GenBank:NM 001126258) [48]. Because these genes are single copy, a correct clustering should identify unigenes orthologous to them as being related. PGD and SNF each had hits to a single contig in the *E. propertius *unigene set (covering 30% and 52% of PGD and SNF, respectively); neither of these contigs were clustered with any other unigenes. The TPI gene had a hit to a contig (covering 56% of TPI) that also was clustered with one other singleton. The other genes, CAD and AATS, had no hits in the *E. propertius *unigene set.

For *P. zelicaon*, the PGD gene had hits to three contigs (together covering 9% of PGD); these were clustered together along with one other singleton. The TPI gene hit a single contig (covering 65% of TPI) that also was clustered with one other contig. The AATS gene hit three contigs representing two full clusters (covering 45% of AATS). The SNF and CAD genes had no hits to the *P. zelicaon *unigene set.

To investigate the absence of CAD and AATS for *E. propertius *and CAD and SNF for *P. zelicaon*, we searched for evidence of these genes in the *M. cinxia *EST dataset [11]. Of the 595,541 uncleaned *M. cinxia *ESTs, 35 hit SNF, 75 hit AATS, and 1 hit CAD. Thus, although these genes appear to be expressed in a lepidopteran larval transcriptome, they appear to be present at low levels in EST collections, particularly for CAD.

#### Metatranscriptomic Contamination

Because material was sampled from whole larvae, we expect some unigenes to represent species other than *E. propertius *and *P. zelicaon*. Of the 15,555 *E. propertius *unigenes with no hits to *D. melanogaster*, *B. mori*, *H. erato*, *M. cinxia*, or *P. xuthus*, 90 had hits to other Metazoa (63 Insecta; see Methods). 69 *E. propertius *unigenes hit Viridiplantae, 16 hit Bacteria, 5 hit Fungi, and 12 unigenes hit species in various other kingdoms.

Of the 12,941 *P. zelicaon *unigenes with no hits to the five species mentioned above, 165 hit Metazoa (132 Insecta). 56 hit Viridiplantae, 22 hit Bacteria, 41 hit Fungi, and 12 hit other kingdoms. For both species, the bacterial hits included one singleton which hit to *Wolbachia *(of *D. melanogaster*, e-value 2e-6 for *E. propertius *and 2e-15 for *P. zelicaon*).

### Genetic Diversity

#### SNP Detection

Single Nucleotide Polymorphisms (SNPs) were identified by analyzing the multiple alignments produced during the assembly process using both a "loose" criterion to maximize the discovery of rare alleles, and a "strict" criterion to minimize the possibility of false positives due to sequencing error (see Methods).

Table 3 shows SNP counts and other statistics using these two criteria. For both criteria, *E. propertius *had a slightly higher percentage of transversions than *P. zelicaon *(45% and 44% vs. 43% and 42%, respectively). These transversion percentages are between those found for *B. mori*, 37.5% [49], and *D. melanogaster*, 51.9% [50].

**Table 3:** SNP discovery statistics. Loose criterion: non-gap consensus in the multiple alignment, minority nucleic allele found in at least two ESTs. Strict criterion: non-gap consensus in the multiple alignment, minority nucleic allele found in at least 25% of ESTs covering the position, at least 6x coverage at the position. Non-synonymous and synonymous SNPs were counted via best BLAST hits to *B. mori*.

	SNPs	Transversions	Transitions	Non-Syn	Syn	Contigs With SNPs
Loose SNP Criterion						

*E. p*.	94,783	42,719	52,064	5,341	20,520	8,042 (7.52 Mbp)
*P. z*.	127,004	54,934	72,070	10,193	36,170	8,888 (7.97 Mbp)

Strict SNP Criterion						

*E. p*.	36,014	15,895	20,119	1,648	8,273	6,298 (6.11 Mbp)
*P. z*.	62,655	26,545	36,110	4,527	18,509	7,223 (6.75 Mbp)

For *E. propertius*, strict criterion SNPs were found in 6,298 contigs, comprising 6.11 Mbp of sequence. Thus, we estimate at least 36,014 SNPs in 6.11 Mbp or 5.89 SNPs per 1,000 bases for *E. propertius*. Similar calculations for *P. zelicaon *discover at least 9.28 SNPs per 1,000 bases. In comparison, Vera et al. estimated 12.6 SNPs per 1,000 bases in probable coding regions of the *M. cinxia *transcriptome using similar source data and similar conservative criteria for identifying SNPs [11].

We also can label SNPs that appear in putative coding regions (as found by BLAST against *B. mori*, see Annotation) as non-synonymous or synonymous (Table 3). Strict criterion SNPs occurred in 2,067 putative *E. propertius *coding regions, representing 1.52 Mbp of sequence. Thus, we estimate at least 1,648 × 10^3^/1.52 × 10^6 ^≈ 1.08 non-synonymous SNPs and 8,273 × 10^3^/1.52 × 10^6 ^≈ 5.44 synonymous SNPs per 1,000 base pairs in coding regions of the *E. propertius *transcriptome. For *P. zelicaon*, strict SNPs occurred in 3,384 putative coding regions representing 2.19 Mbp of sequence, for an estimate of 2.09 non-synonymous and 8.56 synonymous SNPs per 1,000 base pairs in coding regions of the *P. zelicaon *transcriptome.

#### Celera Variant Detection

Recent versions of the Celera assembler cluster co-occurring SNPs and indels together into "variants"--polymorphisms that may include more than a single nucleotide but yet are not large enough to be considered haplotypes. These variants also inform the Celera assembly process, so that chimeric contigs containing nearby allele combinations not found in nature are avoided [51]. By default, variants are identified by grouping polymorphisms together so long as a stretch of at most 11 non-polymorphic sites occur between them, and each allele is supported by at least two reads. Quality values are also used.

Not counting single nucleotide length variants (SNPs surrounded by at least 11 non-polymorphic sites), the assembly produced 6,775 variant regions in 3,697 *E. propertius *contigs, with an average length of 3.43 bp (maximum 41 bp). The average number of variants per region was 2.93, with a maximum of 17, consistent with the maximum number of genotypes sequenced. In this case, the largest variant region of 41 bp was also the region with the most number of variants, 17. The vast majority of ESTs (428/475) in this region supported a single variant, with the second most frequent variant occurring in only 9 ESTs.

For *P. zelicaon*, there were 5,636 variant regions in 3,494 contigs, with average length 2.90 bp (maximum 27 bp--a large indel with only two variants). The average number of variants per region was 2.42, with a maximum of 12 (a region of length 6 bp). This large number of variants is inconsistent with the fact that only 10 genotypes were sequenced, and may indicate paralog collapse during assembly or sequencing error. Alternatively, it is possible that one or more of the *P. zelicaon *mothers was fertilized by more than one male, resulting in more genotypes present in the data than female lines [52,53]. This is the only variant region for *P. zelicaon *with more than 10 variants.

#### *β *Parameter

Because ESTs were sequenced from a number of genotypes and because assembly coverage varies among contigs, standard measures of nucleotide diversity such as *θ *[54] can not be calculated. Instead, we consider a relative measure of nucleotide diversity *β*_*t *_developed by Novaes et al. [12], defined for contigs with average coverage at least 2×. (Note that even contigs with 2× average coverage can have regions of locally high coverage where SNPs can be found.) However, for all that follows, we compute *β *statistics only for those contigs with at least 6× average coverage to avoid biases caused by contigs that represent diverse sequences but are expressed at low levels. For contigs that also have a *B. mori *best hit (see Annotation), we can compute *β*_*n*_, a diversity estimate for non-synonymous sites, and *β*_*s*_, a diversity estimate for synonymous sites [12]. *β*_*t*_, *β*_*n*_, and *β*_*s *_are formally defined as follows:

In the above, *S*_*t *_is the number of SNPs in the contig (using the strict SNP criterion; see Annotation), *S*_*n *_is the number of non-synonymous SNPs in BLAST annotated putative coding regions, *S*_*s *_is the number of synonymous SNPs in putative coding regions, *L*_*t *_is the total length of the contig, *L*_*c *_is the length of the putative coding region, *D *is the average coverage depth, and *H*_*n *_is the *n*^*th *^harmonic number. Table 4 shows average and median values of *β*_*t*_, *β*_*n *_and *β*_*s *_amongst contigs with at least 6× coverage for both species.

**Table 4:** *β *parameter statistics for contigs. Statistics for *β*_*t*_, *β*_*s*_, and *β*_*n *_are over all contigs for which those values are individually defined and contig average coverage is at least 6×. SNPs used in calculation are identified using the strict SNP criterion (see Annotation).

	*β*_*t*_		*β*_*n*_		*β*_*s*_	
		*Median*		*Median*		*Median*
*E. p*.	2.44 × 10^-3^	1.83 × 10^-3^	1.00 × 10^-3^	0.73 × 10^-3^	1.93 × 10^-3^	1.52 × 10^-3^
*P. z*.	3.55 × 10^-3^	1.29 × 10^-3^	1.29 × 10^-3^	0.92 × 10^-3^	2.88 × 10^-3^	2.31 × 10^-3^

Novaes et al. note that because *β *statistics are conditioned on coverage depth rather than the actual number of haplotypes sampled, care must be taken in comparing to more traditional diversity estimates such as *θ *[12]. However, these statistics do enable the study of relative genetic diversity within each transcriptome [12], and may speak to comparative diversity estimates for *E. propertius *and *P. zelicaon *if allele sample rates are equal (which is not the case; nevertheless, see Discussion).

The average coverage for *E. propertius *contigs in the top 1% of *β*_*t *_was relatively low at 8.8× (even considering the fact that this is computed only over contigs with at least 6× coverage). The average *β*_*t *_for *E. propertius *contigs in the top 1% of coverage also was low at 0.68 × 10^-3^. For *P. zelicaon*, average coverage in the top 1% of *β*_*t *_was 10.64×, and the average *β*_*t *_in the top 1% of coverage was 1.29 × 10^-3^.

Thus, for both species, very diverse contigs tend to have less than or near average coverage; conversely, highly covered contigs have low diversity. In the presence of large scale paralog collapse, we would expect to see many contigs with high coverage and high *β*, which we have not found.

## Discussion

For *E. propertius*, the large sequences produced by the 454 FLX Titanium allowed for the formation of a 14.6 Mbp assembly from 176 Mbp of EST sequence, with average contig coverage of 10× and average contig length of 753 bp. Similar results were obtained for *P. zelicaon*.

Comparisons to *Bombyx mori *suggest that our final assemblies are of high quality. Because *β*_*t *_was generally low for highly covered contigs, and nearly all variant regions had fewer variants than the number of genotypes sequenced, we see little evidence for over-assembly and paralog collapse. Further, the fact that amongst the assemblies tested we have not seen a point of diminishing returns in terms of average ortholog hit ratio (Table 1) suggests that even more aggressive assemblers may produce more accurate assemblies for such diverse datasets.

Clustering results and comparison to the *P. xuthus *mitochondrial genome indicate the presence of ribosomal RNA in at least the *P. zelicaon *dataset. Although mitochondrial genes (e.g. ND5 and ATP6) are polyadenylated and appropriately found in our datasets, ribosomal RNAs are not, and hence should be considered contamination. While such unigenes can easily be filtered after assembly, the fact that many of these were clustered via hits to a protein predicted dataset (for *H. erato*) highlights the need for well annotated and curated reference datasets.

Clustering results also reveal that greater than 90% of unigenes had no similarity with other unigenes, indicating thorough assemblies. We searched for five single copy genes present in *B. mori *[48]. For those *E. propertius *homologues we found, assembly of unigenes was fairly complete (only one contig associated with each gene) and clustering was accurate (only one cluster contained an extra singleton). For *P. zelicaon*, assembly was less complete (multiple contigs per gene), although in only one case were contigs split across two clusters. Coverage of found genes was around 50%, with the exception of the low coverage for PGD. For both organisms, no evidence was found for two of the five genes; based on similar analysis for *M. cinxia *ESTs, this appears to be the result of low expression in larval samples.

Determining the breadth of coverage of the transcriptomes is difficult, given how little is known about butterfly genomes. Unigenes for both *E. propertius *and *P. zelicaon *hit ~ 8 K (of ~ 14.5 K total) unique *B. mori *predicted proteins. For both species, at least 9 K unigenes hit one of *B. mori*, *H. erato*, or *D. melanogaster*. Excluding the largest clusters, these hits were distributed roughly evenly between high and low coverage contigs and singletons, supporting previous studies suggesting that singletons and low coverage contigs are biologically valuable [14]. Gene ontology term analysis reveals that 24 high level categories are present for both species in levels similar to that for *B. mori*. Thus, although we cannot speculate on how many transcripts exist in these transcriptomes, they appear to be sampled broadly.

As expected in whole larval samples, we identified unigenes representative of plants, bacteria, fungi, and other non-lepidopteran sources [11]. Interestingly, a single EST from both species hit to *Wolbachia*, a symbiotic bacteria known to affect population dynamics and hypothesized to be present in *E. propertius *populations [23].

Vera et al. compared unigene length to length of the best hit protein to estimate completeness of transcript discovery [11]. Unfortunately, this also includes untranslated regions in unigenes, artificially inflating the desired measure. Our alternative, the ortholog hit ratio, provides a more conservative estimate of the effectiveness of gene discovery and speaks to assembly quality. Greater than 5% of unigenes had a ratio > 0.8 and greater than 10% of unigenes had a ratio > 0.5 for both species. We conclude that at least ten percent of our putative *B. mori *orthologs capture approximately 50% of their corresponding silkworm genes.

The effects of alternative splicing on the ortholog hit ratio depend on the abilities of the assembler, as well as on whether the reference *B. mori *protein set contains orthologs to alternatively spliced transcripts. Since many assemblers split contigs at ambiguous or repetitive regions [55], alternative splicing will likely result in a low ortholog hit ratio for the alternative version, even if the alternative ortholog exists in the reference dataset. If the alternative ortholog does not exist in the reference dataset, either the alternative segment will match a subregion of the original transcript form (resulting in a low ortholog hit ratio), or there may be no hit at all (resulting in an undefined ortholog hit ratio). Since these issues serve to reduce ortholog hit ratios rather than inflate them, the conservativeness of the ortholog hit ratio approach is preserved.

While estimating genetic diversity accurately (e.g. computing *θ *[54]) is difficult given the essentially unknown number of natural alleles contributing to the population sample for each contig, some relative comparisons between species should be possible. For example, using a SNP calling criterion similar to our strict criterion and a source dataset similar to ours, Vera et al. estimate 12.6 SNPs per 1,000 base pairs in the *M. cinxia *transcriptome [11], whereas we estimate 5.89 SNPs/1,000 bp for *E. propertius *and 9.28 SNPs/1,000 bp for *P. zelicaon*.

While Novaes et al. caution against comparing *β *diversity estimates across assemblies--as they depend on sequencing depth, SNP calling criteria, and other factors--the *E. propertius *and *P. zelicaon *datasets were collected and processed in nearly identical fashion. Average *β *statistics were higher in *P. zelicaon *than in *E. propertius*, despite smaller sample sizes for *P. zelicaon *(owing to the relative difficulty of specimen collection). These comparative diversity results, both in terms of raw SNP counts per 1,000 bases and *β*, support previous findings that overall genetic polymorphism is higher for *P. zelicaon *than *E. propertius *[22].

As was found for *Eucalyptus grandis*, the *β *distributions are all right-skewed (Table 4), suggesting purifying selection for the majority of genes [12]. *β*_*t *_is slightly negatively correlated with the number of species hit (0,1,2, or 3 of *B. mori*, *H. erato*, and *D. melanogaster*) for *E. propertius*, suggesting that lineage and species specific genes are more diverse for *E. propertius *(*r *= -0.154, *p *< 0.0001). A similar, but weaker and non-significant, trend is found for *P. zelicaon *(*r *= -0.0254, *p *< 0.054).

As has been noted before [38], different assembly programs can produce very different results, as seen in Table 1. While none of the assembly programs currently in widespread use are designed for ecoinformatics, Liang et al. have suggested that CAP3 is the best choice for ESTs [56]. However, Liang et al. did not consider the Celera Assembler, and our results suggest that new versions of the Celera Assembler may be more appropriate for data containing a diversity of genotypes.

For further comparison, we also assembled the *E. propertius *and *P. zelicaon *EST sets with the recently released Newbler assembler version 2.3 (Roche 454 Life Sciences), which has options specifically for transcriptome data. For *E. propertius*, Newbler produced 19,110 contigs of average length 637 bp and 36,848 singletons with average (uncleaned) length 314 bp. For *P. zelicaon*, 25,336 contigs of average length 730 bp and 20,926 singletons of average (uncleaned) length 297 bp were produced. Newbler version 2.3 also produces a set of sequences known as "isotigs," arrangements of contigs meant to represent splice forms (similar to [55]). For *E. propertius*, 11,677 such isotigs with average length 1,238 bp were produced. 17,520 isotigs of average length 1,309 bp were produced for *P. zelicaon*.

Another factor in successful transcriptome assembly is the sequencing technology used. In our application, the 454 Titanium chemistry sequencer produced average read lengths of about 400 bp. In contrast, the older 454 GS-20 platform used by Vera et al. produced reads averaging 110 bp for the *M. cinxia *transcriptome [11]. To assess the effects of sequencing technology, we obtained *M. cinxia *ESTs from the Sequence Read Archive (SRA: SRR000670 and SRR000671) and cleaned and assembled them similarly to our datasets. (The original assembly by Vera et al. used SeqmanPro, a proprietary assembler from DNAStar.) After cleaning, 575,313 ESTs of average length 100 bp remained. Our assembly produced 34,921 contigs (average length 141 bp), and 27,468 singletons (average length 81 bp). The fact that this assembly size is different from that produced by Vera et al. indicates that reanalysis of data may be important as new bioinformatics tools and assemblers become available.

Comparison between the above *M. cinxia *assembly and that for *P. zelicaon *or *E. propertius *is complicated by multiple factors. First, these are different species with different patterns of diversity and expression. Second, even though the number of cleaned ESTs is similar, the shorter read lengths for *M. cinxia *ESTs provide less total sequence data, affecting the number of contigs obtained. Nevertheless, the similar aspects of these datasets (including that they were all sourced from several individuals) does suggest that longer read lengths can improve assembly quality.

## Conclusion

We reported larval transcriptome sequences and assemblies for butterflies of ecological importance: *Erynnis propertius *(Lepidoptera: Hesperiidae) and *Papilio zelicaon *(Lepidoptera: Papilionidae). As the immediate aim was construction of a microarray enabling comparison of transcribed genes under alternative climate treatments and of populations of differing geographic locations, steps were taken to maximize gene discovery within the larval stage.

Long read lengths produced by the 454 FLX Titanium sequencing platform and experimentation with assembly techniques produced high quality assemblies with few singletons. Over ten percent of putative *B. mori *orthologs in *E. propertius *and *P. zelicaon *cover at least 50% of the corresponding silkworm gene, as measured by ortholog hit ratio. Gene ontology annotation suggests that transcripts were broadly sampled, and comparisons with *Bombyx mori *and other related model species indicate that many genes were found--both species had hits to over 50% of the *B. mori *protein dataset.

Although the ortholog hit ratio does not consider the effects of alternative splicing (unless alternative splice forms also exist in the reference dataset), it appears to be an excellent method for the comparative assessment of assemblies. Using this measure, as well as simpler measures such as contig and singleton count, we found the Celera Assembler to be an effective tool for handling population-level datasets, particularly when custom parameters are used.

454 sequencing and assembly has proven an effective platform for SNP discovery [3,11-14,41,57]. Variant regions detected with the Celera Assembler may prove useful for population-level studies, further supporting Celera Assembler for this type of data. Significantly, the discovery of ~ 36 K high quality SNPs for *E. propertius *and ~ 62 K SNPs for *P. zelicaon *will facilitate future studies of population structure and genetic causes of functional differences already found between populations [22,24,25].

## Methods

### Rearing and RNA Isolation

Eggs laid by adult *E. propertius *and *P. zelicaon *females were hatched under conditions characteristic of native habitats in a greenhouse and then moved to Conviron growth chambers at the University of Notre Dame. Multiple individuals of each larval instar were collected through the final instar before pupation [20]. Individuals of the 2^nd^, 3^rd^, and 4^th ^instars (*E. propertius*) and 3^*rd *^and 4^th ^instars (*P. zelicaon*) were exposed to a heat stress of 35 degrees for 60 minutes and a cold stress of 0 degrees for 120 minutes. Individuals in the 5^th ^and 6^th ^instar of *E. propertius *and 3^rd ^and 5^th ^instars of *P. zelicaon *were exposed to a desiccation agent (silica gel) for 120 minutes. In addition, some of the collected larvae of *P. zelicaon *were fed *Petroselinum crispum *and others were fed *Lomatium utriculatum*. The former contains higher concentrations of linear furanocoumarins, a defensive compound against herbivores, than the latter [58]. After treatment, larvae were frozen in liquid nitrogen and stored at -80°C.

Whole-body RNA from these frozen individuals was extracted using an RNA Easy kit (QIAGEN Inc.) over a period of two months. Prior to library construction, pooling was done by adjusting sample contributions to equimolar amounts of total RNA per individual.

### Library Construction and 454 Sequencing

Library construction was performed by Express Genomics, Inc. (Frederick, Maryland, USA). Poly(A)+RNA from the *E. propertius *and *P. zelicaon *total RNAs was isolated by two rounds of oligo(dT) selection with oligo(dT) coated magnetic particles (Seradyn, Inc.).

From the poly(A)+RNA mRNA, cDNA libraries were constructed by using an oligo dT primer-adapter containing a *Not *I site and Moloney Murine Leukemia Virus Reverse Transcriptase (M-MLV RT) to prime and synthesize first strand cDNA. This process includes only one round of reverse transcription. After the second strand was synthesized, the double stranded cDNA was size fractionated (> 1.4 kb) and cloned directionally into the *Not *I and *Eco *RV sites of the pExpress 1 vector. From one bulk ligation (300 ng of pExpress 1 vector, *Not *I-*Eco *RV cut, and 120 ng of *Not *I digested cDNA per 120 *μ*l of ligation), followed by electroporation into T1 phage resistant *E. coli*, primary clones were produced.

Normalized cDNA libraries were produced from the primary cDNA libraries. Biotinylated driver RNA produced from the T7 RNA polymerase promoter and single-stranded (ss) target DNA produced from the F1 ori were hybridized to each other at a low Cot (concentration of driver times the time of hybridization) value. The RNA:DNA hybrids were removed by phenol extraction and the remaining ss target DNA was converted to double-stranded DNA (dsDNA) with a repair oligo and *Taq *DNA polymerase. After electroporation of the dsDNA into T1 phage resistant *E. coli*, primary clones were produced.

The *E. propertius *and P. *zelicaon *normalized library DNAs were digested with *Not *I and *in vitro *RNA transcripts were produced using the SP6 RNA polymerase promoter. Then, first strand cDNA was made from these transcripts using a modified primer adapter that reduces the size of the poly A sequence (to about 20 As). After the second strand was synthesized, the double stranded (ds) cDNA was blunt ended and size fractionated. This ds cDNA was resuspended in TE, pH 8.0, to between 110-125 ng/ml.

The pooled sample for each species was run on one-half of a plate on a 454 FLX Titanium machine at the Research Technology Support Facility at Michigan State University (East Lansing, Michigan, USA). EST sequences and quality scores are available from the National Center for Biotechnology Information (NCBI) Sequence Read Archive, accessions SRR035432.1 (*E. propertius*) and SRR035433.1 (*P. zelicaon*).

### Cleaning and Assembly

ESTs were cleaned and vector trimmed with SeqClean [36] using both the NCBI Univec-Core database and the pExpress 1 vector (using the -v option to look for vector contamination), and the ESTs were scanned for *E. coli *(strain K-12, substrain MG1655) contamination (using the -s option for contamination screening). The default minimum read length cutoff of 100 bp was used (using a larger, 200 bp cutoff did not drastically alter the singleton length distributions seen in Figures 1(a) and 1(b), data not shown).

Cleaned ESTs were assembled with CAP3 [37] and Celera Assembler [39,51], using two parameter sets each. Default CAP3 settings include -p 90 -h 20; the custom parameter settings used were -p 85 -h 90. The CAP3 -p option specifies overlap percent identity cutoff, while the -h option specifies the maximum alignment overhang percentage. The recommended settings for Celera Assembler on 454 FLX Titanium chemistry are as follows: overlapper = mer (use a seed and extend overlap algorithm), unitigger = bog (use a best overlap graph approach for building unitigs), utgErrorRate = 0.03 (the error rate above which the unitigger discards overlaps), doOverlapTrimming = 1 (perform overlap based trimming) [40]. The final Celera assemblies used the following parameters: overlapper = mer, unitigger = bog, utgErrorRate = 0.07, ovlErrorRate = 0.07, cnsErrorRate = 0.07, doOverlapTrimming = 1. The parameter ovlErrorRate specifies an error rate above which the overlap module of the Celera Assembler will not report overlaps. Thus, the custom settings for both CAP3 and Celera Assembler allow for more tolerance of sequence divergence in assembling contigs.

### Gene Ontology Terms Distribution

Gene ontology terms were assigned to unigenes and *B. mori *predicted proteins by via BLAST against the non-redundant nucleotide database NR (obtained September 20, 2009) and analyzing the results using the Blast2GO tool [59]. These terms were mapped to high level gene ontology categories with CateGOrizer [60], using the "Aqua" categorization.

### SNP Detection

Single nucleotide polymorphisms were identified by independently analyzing each column of the multiple alignments produced for contigs during the assembly process. Both the "loose" criterion, designed to maximize the discovery of rare alleles, and the "strict" criterion, designed to minimize the possibility of false positive identification, required that the consensus position not be a gap in the multiple alignment, and that there be at least two distinct nucleotide alleles present for that column.

The loose criterion required only that each of the two most common alleles be found in at least two ESTs. The strict criterion required that the minority allele (the second most common nucleic allele) be found in at least 25% of the ESTs covering that position, and that the total coverage at that position be at least 6×. The loose SNP criterion is prescribed by Long et al. [61] as an effective method for SNP discovery in EST projects and is used as a secondary criterion by Barbazuk et al. [3]. The strict criterion is very similar to that used by Vera et al. [11]. Because it requires 25% minority allele coverage in highly covered areas, it is less sensitive to false positives than the loose criterion.

### Metatranscriptomic Contamination

Unigenes not having hits to one of *D. melanogaster*, *B. mori*, *H. erato*, *M. cinxia*, or *P. xuthus *(see Annotation) were compared against the NCBI Non-Redundant protein set NR (obtained September 20, 2009) using BLASTX (cutoff 1e-5). Best hits were parsed using MEGAN version 3.8 [62]. Assigned hits were counted at the kingdom taxonomic level, with the Min Support option set to 1 (such that every hit with bitscore greater than the default cutoff of 35.0 is assigned to a taxa).

### Clustering

Unigenes were clustered based on BLAST similarity to other unigenes of the same species, *B. mori*, and *H. erato *protein databases. Each unigene, *B. mori *protein, and *H. erato *protein was considered as a vertex in a graph (representing sequence similarity between elements of the datasets). Each unigene was connected to its best BLASTN unigene match, best BLASTX *B. mori *hit, and best BLASTX *H. erato *hit (*e *< 1e-5 cutoff) by an undirected edge in the graph. Clusters of unigenes were those present in the same connected component of the graph--that is, unigenes that are reachable from each other by following a path in the graph [63]. For example, if unigene A had a hit to unigene B, and unigenes B and C both had hits to a *B. mori *protein X, then A, B, and C would be considered a cluster.

## Authors' contributions

STO performed most of the bioinformatics analysis and drafted the manuscript. JDK supervised fieldwork, specimen collection, and cDNA sequencing. RDC helped assemble and analyze the data. NFL participated in design, data analysis, assembly validation, and drafting the manuscript. SJE helped conceive the study, coordinated analysis, and helped draft the manuscript. JJH conceived the study and helped coordinate and draft the manuscript. All authors read and approved the final manuscript.
